# Person-centred innovations for family medicine and COVID-19 in Ghana: A short report

**DOI:** 10.4102/phcfm.v13i1.3016

**Published:** 2021-08-23

**Authors:** Daniel O. Darko

**Affiliations:** 1 Medical Department, Nyaho Medical Centre, Accra, Ghana

**Keywords:** family medicine, primary health care, COVID-19, eHealth, Nyaho Medical Centre

## Abstract

Countries around the world have mobilised health, social, and economic resources to control the coronavirus disease 2019 (COVID-19) pandemic since its discovery in December 2019. Primary care as a frontline of many health systems, played a huge role in the management of the current pandemic. This is a short report by Dr Daniel Osafo Darko, a family medicine resident at the Nyaho Medical Centre in Accra, Ghana. It details some contributions of Nyaho Medical Centre to the fight against COVID-19 in Ghana by providing clinical care, using eHealth approaches.

## Introduction

Since December 2019 until now, the world has been fighting against a novel coronavirus disease referred to as coronavirus disease 2019 (COVID-19). Countries around the world have since then mobilised health, social, and economic resources to curb the spread of the disease and improve the health system capacities. Ghana reported its first two cases of COVID-19 on March 12, 2020 and both the cases were imported by Turkish and Norwegian nationals.^1^ The Ghana Health Service (GHS) announced the set-up of a national COVID-19 taskforce to lead and coordinate surveillance, early detection and treatment efforts.^2^ The Mechanisms devised by the GHS relied on tracing and testing all potential cases, making Ghana to have one of the highest testing rates per 1000 persons in Africa during the early months of the pandemic.^3^ There were concerted efforts by the President of the Republic, GHS, Ministry of Health (MOH), and the national COVID-19 taskforce to manage the situation using different directives including social distancing measures, closing down of educational institutions, restricted entry for foreign nationals, and the mandatory wearing of facemasks in public.^4^

**FIGURE 1:**
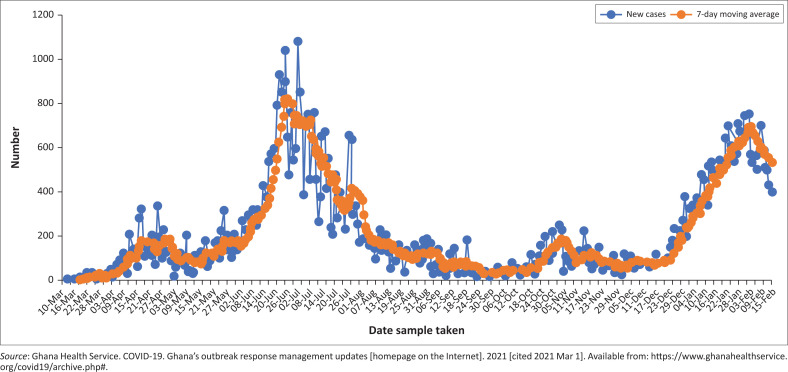
Distribution of coronavirus disease 2019 cases and 7-day moving average in Ghana by date sample taken, March 2020 – February 2021.

Despite these efforts, COVID-19 rapidly moved to community transmission. Figure 1 shows the distribution of COVID-19 cases from March 2020 to February 2021. To effectively combat the pandemic in Ghana, collaboration by all key stakeholders and deployment of innovative approaches were crucial.

## Nyaho Medical Centre

I am a final year Family Medicine resident at Nyaho Medical Centre (NMC). In the medical school, I aspired to go into a different specialty in medicine, but my work in NMC as a medical officer after internship exposed me to family medicine and I am really enjoying the experience. The NMC is a premiere private medical centre at the Airport Residential Area in Accra, Ghana with a rich 51-year history of providing primary and secondary care to the middle- and high-income population in Accra and beyond. The NMC received accreditation from the Ghana College of Physicians and Surgeons in 2016 for residency training in Family Medicine and currently has 10 residents in the programme. As a family medicine resident, my schedule is dedicated to rotations in different health facilities in Accra and at least one 12-h shift at NMC every week.

## Innovations for coronavirus disease 2019

Within a matter of days after the first cases of COVID-19 were identified in Ghana, NMC recorded several cases at our outpatient department. By the end of June 2020, NMC was designated as a COVID-19 treatment facility and opened the first private polymerase chain reaction (PCR) laboratory in the country. The NMC also adapted its operations by creating innovative ways of meeting patient needs including the use of eHealth to deliver patient care and education.

A mobile number was dedicated as a COVID-19 hotline and advertised from the middle of March 2020 mainly to meet the demands of the time. This coincided with an arrangement with Noguchi Memorial Institute for Medical Research to test samples collected at Nyaho Medical Centre. A doctor was subsequently scheduled daily to operate the NMC COVID-19 hotline and also call the patients who tested positive to provide initial medical advice. As one of the doctors on this schedule, I often managed asymptomatic and mild COVID-19 cases via telephone and arranged for inpatient care in a few cases. Frequently, there were queries made via the hotline pertaining to the anxiety of testing positive, how to deal with family contacts and about the medications needed.

As evidenced elsewhere, effective health information is critical in fighting a pandemic.^6^ The NMC hosted a series of virtual educational sessions to promote evidenced-based learning during the pandemic, via its Facebook live page starting from 24 April 2020 (Table 1). These sessions targeted specific aspects of COVID-19 information including family care, pregnancy, occupational health, and the association with chronic comorbidities like diabetes and hypertension. Each session was one hour long. Together with other family medicine residents, we took time to answer the questions of the viewers and addressed the myths and misconceptions related to the pandemic. There were growing misconceptions and misinformation in the public regarding the COVID-19 pandemic, which needed to be addressed by health professionals. Our COVID-19 hotlines also provided additional insights into the key concerns and questions which were worth addressing. All sessions were advertised in audio-visuals and in texts on all our social media platforms.

**TABLE 1:** Date, topic, presenter, number of views as captured on Facebook and links to the educational videos.

	Date	Topic	Presenter	Views	Link
1	24th April, 2020	COVID-19	Dr. Daniel Osafo Darko	1.2 K	https://fb.watch/3UBOlewJ5G/
2	8th May, 2020	COVID-19 & pregnancy	Dr. Daniel Osafo Darko	8.6 K	httos://fb.watch/3UBQhZZ6FT/
3	22nd May, 2020	COVID-19 & your family	Dr. Daniel Osafo Darko	818	https://fb.watcht3UCltQODiSo/
4	19th June, 2020	COVID-19 & your occupational health	Dr. Daniel Osafo Darko	554	httos://fb.watch/3UC2HE9kzU/
5	3rd July, 2020	COVID-19 & your mental health	Dr. Ruth Osei Odom	454	https://fb.watch/3UC8OqakeDi/
6	16th August, 2020	Managing diabetes Pt. 1	Dr. Daniel Osafo Darko	560	https://fb.watch/3UCcxNmCDS/
7	6th September, 2020	Managing diabetes Pt. 2	Dr. Daniel Osafo Darko	334	https://fb.watch/3UChO8FNJb/
8	20th September, 2020	Managing hypertension Pt. 1	Dr. Nathan Kwablah	370	https://fb.watch/3UCvwTwkpl/
9	4th October, 2020	Managing hypertension Pt. 2	Dr. Nathan Kwablah	350	https://fb.watch/3UCyF1rhHg/
10	25th October, 2020	Breast cancer awareness	Dr. Nathan Kwablah & Dr. Ewuradjoa Kankam-Yeboah	220	https://www.instagram.com/tv/CGxj8pdJpxP/?igshid=2ohpc5biol9l

COVID-19, coronavirus disease 2019.

True to Nyaho Medical Centre’s mission of transforming the lives of patients by surpassing expectations in healthcare and inspiring hope for a better Africa, the hospital was widely complimented by the public for their immense efforts during the current pandemic. This, in turn, increased the demand for COVID-19 care from the facility. In June 2020, NMC and Clearspace Labs developed a telemedicine platform (Virtual Care). ‘Virtual Care’ used an encrypted video consultation which greatly enhanced care for people with COVID-19 infection as compared to the previously used telephone consultation. We employed video conferencing technology. In addition to the greater convenience it provides, the possibility of seeing your doctor rather than just hearing his or her voice over the telephone, gave patients trust and improved the doctor-patient interaction. Virtual care was advertised on our website and social media platforms and patients used the link provided to book and pay for a consultation via an electronic wallet service (mobile money) at their convenience. Together with another family medicine resident, we managed 36 patients via the telemedicine platform between 30 June 2020 and 31 August 2020. Most of these patients had two virtual consultations within the 14 days of isolation and their medications were delivered to them at home. After each consultation, the pharmacy liaised with the patient for home delivery using a dispatch rider. This encouraged patients to continue to safely self-isolate at home thereby limiting the spread of the disease. Additionally, I provided consultations through Virtual Care for patients who needed refills of medications for chronic conditions or who had other health concerns, but who were afraid to visit a health facility. Patients were encouraged to complete a simple registration form via the hospital’s website to schedule appointments with doctors. Typically, an assigned doctor who was not attending to patients physically present at the hospital, attended to virtual consultations in real time as appointments were made. The online consultations were initiated by the patient using the advertised link.

Understanding the challenges our country faces with digital literacy, the hotlines offered an alternative for the people who could not otherwise access the internet or complete their appointment on our website, to call in and have their COVID-19 related concerns addressed. An assigned doctor also operated this hotline. Coronavirus disease 2019 patients who were asymptomatic or with mild symptoms were usually managed via the hotline after initial diagnosis is confirmed with a PCR.

## What did we learn in family medicine?

Fighting a virus that was new to us meant we had to do new things to win. As family physicians, we were innovative and employed different strategies and tools to maintain healthcare access whilst fighting a pandemic. The information provided through the virtual educational sessions helped to reject the myths surrounding the virus and empowered individuals to adhere to the precautions that limit the spread of COVID-19. The ‘virtual care’ and telephone consultations helped to manage infected patients at a time when there was so much uncertainty regarding COVID-19 and care was difficult to access because of the associated panic. Health promotion activities and the adoption of virtual consultations are the key areas where family medicine can continue to strengthen to improve healthcare access in Ghana and Africa. Major logistical challenges are expected with the problems regarding electricity, fast, affordable and reliable internet, and digital literacy still existing. The NMC is a private facility, which makes it easier to timely implement innovations. The pandemic has taught us the importance of reducing redundant political bureaucracy, which will be crucial in implementing similar strategies in government health facilities.

## Conclusion

This is the beauty of family medicine – providing care for all, irrespective of their condition and influencing the health outcomes at the primary care level, one patient at a time in the context of his family. The strong presence of family physicians and family medicine residents in NMC was crucial in delivering on these innovations as many of us were involved in the planning and execution of healthcare. As the pandemic continues, more innovative and context-specific strategies will be necessary to fight against the COVID-19 pandemic at a global scale.
